# Inactivation of mammalian spermatozoa on the exposure of TiO_2_ nanorods deposited with noble metals

**DOI:** 10.1186/s40543-022-00366-x

**Published:** 2023-01-26

**Authors:** Young-Joo Yi, Love Kumar Dhandole, Dong-Won Seo, Sang-Myeong Lee, Jum Suk Jang

**Affiliations:** 1 Department of Agricultural Education, College of Education, Sunchon National University, 255 Jungang-Ro, Suncheon, 57922 Republic of Korea; 2 Division of Biotechnology, College of Environmental and Bioresource Sciences, Jeonbuk National University, 79 Gobong-Ro, Iksan, 54596 Jeonbuk Republic of Korea; 3 Department of Vaccine Development, Gyeongbuk Institute for Bio Industry, Andong, 36618 Republic of Korea; 4 Laboratory of Veterinary Virology, College of Veterinary Medicine, Chungbuk National University, Cheongju, 28644 Republic of Korea

**Keywords:** TiO_2_ nanorods, Noble metals, In vitro fertilization, NADPH, Embryo development, Au, Ag, Spermatozoa

## Abstract

Titanium dioxide (TiO_2_) nanorods (NRs) are well-known semiconducting and catalytic material that has been widely applied, but their toxicities have also attracted recent interest. In this study, we investigated and compared the toxic effects of TiO_2_ NRs and TiO_2_ NRs loaded with Ag or Au NPs on boar spermatozoa. As a result, sperm incubated with Ag-TiO_2_ NRs showed lower motility than sperm incubated with controls (with or without TiO_2_ NRs) or Au-TiO_2_ NRs. In addition, sperm viability and acrosomal integrity were defective in the presence of Ag-TiO_2_ NRs, and the generation of intracellular reactive oxygen species (ROS) increased significantly when spermatozoa were incubated with 20 μg/ml Ag-TiO_2_ NRs. We discussed in depth the charge transfer mechanism between enzymatic NADPH and Ag-TiO_2_ NRs in the context of ROS generation in spermatozoa. The effects we observed reflected the fertilization competence of sperm incubated with Ag-TiO_2_ NRs; specifically sperm penetration and embryonic development rates by in vitro fertilization were reduced by Ag-TiO_2_ NRs. To summarize, our findings indicate that exposure to Ag-TiO_2_ NRs could affect male fertilization fecundity and caution that care be exercised when using these NRs.

## Introduction

Titanium dioxide nanoparticles (TiO_2_ NPs) are well-known semiconducting and catalytic material and have been widely used for a variety of applications because of their chemical inertness, thermal–physical stability, low cost, and environmental friendliness (Wu et al. 2015). TiO_2_ NPs have shown effective antibacterial activities (Cornish et al. 2000; Sichel et al. 2007; Dhandole et al. 2017, 2018). In particular, incorporation of noble metal (e.g., Ag, Au, or Pt) nanoparticles on the surface of TiO_2_ particles displayed efficient catalytic properties and antibacterial activities (Rupa et al. 2007; Traversa et al. 2000). Zhang et al. (2016) synthesized hetero-nanostructure Au/TiO_2_ nanocomposites has good antibacterial activity against *Escherichia coli* (1 mg photo-catalyst/ml *E. coli* suspension) under visible light and specifically in the dark. Li et al. (2014) proposed an antibacterial mechanism for plasmonic gold NP-modified TiO_2_ nanotubes (foil based) in the dark and found that localized surface plasmon resonance (LSPR) of gold nanoparticles disrupted electron transfer in the membrane respiratory system and caused bacterial death. It hypothesizes that the respiratory proteins of microbial membranes may behave as n-type semiconductors. The physical contact of microbes with Au nanoparticles will result in Schottky barrier formation and Fermi level alignment which result in the facile electrons transfer from microbial membranes to Au nanoparticles and the resultant increase in surface electron density of Au nanoparticles. It assumes that the energy loss may be converted into light energy here and if the light energy is large enough, it can be absorbed to induce the LSPR of Au nanoparticles. The plasmonic hot electrons will flow to the TiO_2_ conduction band and subsequently to the valence band. Thus, the bacterial membrane (work as electron donor) steadily loses electrons in this way and suffers from the reactive oxygen species (ROS)-independent oxidative stress, which finally damages the membrane integrity and induces the cell death.

In addition to its antibacterial activity, recent studies have reported the effects of TiO_2_ NPs including inflammation, apoptosis, reactive oxygen species (ROS) production, and changes in enzyme activity in living cells, and accumulations in organs (Chang et al. 2013; Shi et al. 2013; Czajka et al. 2015). In an examination of the reproductive system, intraperitoneal injection of TiO_2_ NPs affected testis and epididymis in male mice by reducing sperm counts and motility and increasing sperm abnormalities and germ cell apoptosis rates, while effects on livers and kidneys were slight (Guo et al. 2009). When TiO_2_ NPs were administered to female mice over a long time, investigators observed ovarian injury, subfertility, and a low pregnancy rate (Gao et al. 2012), and buffalo spermatozoa treated with TiO_2_ NPs exhibited DNA damage and excessive ROS production (Pawar and Kaul 2014). The potential toxic effects of silver (Ag) and gold (Au) NPs on reproduction relevant cells have been observed to be toxic to spermatozoa in a concentration, size, or dose-dependent manners and have also attracted research attention (Taylor et al. 2015). Incorporating Ag NPs (0.1, 1, 10, and 50 μg/ml) into spermatozoa induced oxidative stress that impaired fertilization and embryonic development of mouse (Yoisungnern et al. 2015). Another study reported that Au NPs reduced sperm motility, and gold particles can penetrate sperm cells, which resulted in fragmentation (Wiwanitkit et al. 2009), while human sperm cultured with Ag NPs at high doses (greater than 250 μM concentrations) showed slightly higher cytotoxicity than Au NPs (Moretti et al. 2013).

In this study, we examined the effects of synthesized crystalline metal oxide TiO_2_ nanorods (NRs) and TiO_2_ NRs loaded with Ag or Au NPs on boar spermatozoa. In vitro fertilization (IVF) using pig oocytes matured in vitro was used to examine the fertilization competence of spermatozoa incubated without NRs, with TiO_2_ NRs, Ag-TiO_2_ NRs, or Au-TiO_2_ NRs by assessing subsequent embryonic development. In addition, we investigated the sperm toxicity mechanism with the help of nicotinamide adenine dinucleotide phosphate (NADPH), which is used as an electron donor, and hypothesized a charge transfer mechanism where living cells lose electrons related to intracellular ROS generation that eventually compromise membrane integrity and led to DNA fragmentation.

## Materials and methods

### Chemical reagents

Commercially available TiO_2_ nanopowder (P25, Degussa) of average particle size ~ 21 nm was used as the starting material. Na_2_HPO_4_ and NaCl were purchased from Kanto Chemicals (Tokyo, Japan) and Junsei (Kyoto, Japan), respectively. Noble metal precursor silver nitrate and gold (III) chloride trihydrate were purchased from Samchun Chemicals (Seoul, Korea) and Sigma-Aldrich (St. Louis, MO, USA), respectively.

Unless otherwise noted, all other reagents used in this study were purchased from Sigma-Aldrich.

### Preparation of TiO_2_ nanorods

The procedure used for synthesizing TiO_2_ NRs was documented in our previous study (Dhandole et al. 2017). Briefly, TiO_2_ NRs were synthesized using a molten salt flux method. In a typical procedure, commercially available TiO_2_ nanopowder (Degussa), NaCl, and Na_2_HPO_4_ were ground together in the ratio 1:4:1 (by weight) to form a homogeneous mixture. This mixture was calcined inside a box furnace at 825 °C for 8 h, cooled to room temperature (RT), washed with DI water to remove water soluble salts, collected by filtration paper (< 5 µm), and rewashed using the same procedure to remove remaining sodium ions. The collected filtrate was dried overnight at 80 °C inside a hot air oven and then finely ground in a mortar.

### Syntheses of noble metal-loaded TiO_2_ NRs

We prepared noble metal-loaded (Ag or Au) TiO_2_ NRs by photo-deposition under a xenon arc lamp (Abet, Japan) at 150 W. Photo-deposition experiments were performed in a Pyrex vessel at atmospheric pressure and ambient temperature. In a typical experiment, we prepared TiO_2_ NR suspensions by dispersing 100 mg of TiO_2_ NR powder in 60 ml of DI water and then adding 10 ml of methyl alcohol. The reactor mixture was ultra-sonicated and stirred for 5 min, and then, 1 wt% aqueous noble metal precursor solution was added dropwise. Methyl alcohol scavenged holes and accelerated the rate of metal reduction during photo-deposition. Aqueous noble metal solutions were prepared by dissolving noble metal precursors in 15 g of DI water in vials and then kept in a cool dark place. These solutions were added to TiO_2_ NR suspensions at a noble metal to TiO_2_ NR weight ratio of 1%, and the reactor suspensions obtained were continuously stirred for 30 min in the dark and then irradiated for 60 min under solar light. The colored precipitates that formed (purple for Au and gray for Ag) were collected by vacuum filtration on filter paper (0.45 µm), washed with DI water, dried at 80 °C overnight before catalyst experiments and characterizations.

### Characterizations of noble metal-loaded TiO_2_ NR catalysts

We performed X-ray diffraction (XRD) structural analysis using a PANalytical X’pert Pro MPD diffractometer equipped with a Cu–K_α_ radiation source (wavelength *K*_α1_ = 1.540598 Å and *K*_α2_ = 1.544426 Å) operated at 40 kV and 30 mA and a scan rate of 0.03° 2*θ* s^−1^ over a 2*θ* range of 5°–80°. Field emission scanning electron microscopy (FESEM) was performed using a SUPRA 40VP unit (Carl Zeiss, Germany) equipped with X-ray energy-dispersive spectrometry (EDS). Transmission electron microscopy (TEM; Jeol JEM-3100F, Tokyo, Japan, at 200 kV) was performed by placing a drop of a sample suspension in ethanol on a standard carbon-coated copper grid. X-ray photoelectron spectroscopy (XPS, Thermo Fisher Scientific, Waltham, MA, USA) using a monochromatic Al–K_α_ X-ray source (*hν* = 1486.6 eV) was used for elemental quantification and to study valence states.

### Catalytic degradation experiment

Degradation experiments were performed in a Pyrex vessel at atmospheric pressure and ambient temperature using orange II sodium salt dye and UV–Vis spectrophotometry (Shimadzu UV-2600 UV–Vis-spectrophotometer) at the maximum dye absorbance wavelength (λ_max_; 484 nm). Briefly, commercial NADPH was mixed with 10 μl aqueous orange II sodium salt dye (pH 7.0) under continuous magnetic stirring. Dye degradation efficiencies were calculated using the following equation:

1 $${\text{Dye degradation efficiency}}\;(\% ) = \left( {1 - \frac{{A_{t} }}{{A_{{0}} }}} \right) \times 100$$

where *A*_0_ is initial absorbance of the dye solution and *A*_*t*_ is dye absorbance during reaction at time *t*.

### Boar sperm preparation and sperm incubation

Liquid boar semen was purchased from a local artificial insemination (AI) center. The diluted semen was stored in a storage unit at 17 °C for 5 days. Stock solutions (1 mg/ml) of TiO_2_ NRs, Ag-TiO_2_ NRs, and Au-TiO_2_ NRs were prepared by suspension in phosphate-buffered solution (PBS) and sonicated for 10 s at 60 Hz (Daihan Scientific, Korea) before use. For the incubation experiments, sperm were incubated in Beltsville thawing solution (BTS; Pursel and Johnson 1976) in the absence or presence of TiO_2_ NRs (controls), Au-TiO_2_ NRs, or Ag-TiO_2_ NRs at final concentrations of 10 or 20 μg/ml, which did not interfere with sperm movement or fertilization, for 2 h at 37.5 °C. All experiments were repeated at least six times.

### Assessment of sperm motility

Sperm motility was quantified using a computer-assisted sperm analysis system (CASA, Sperm Class Analyzer®, Microptic, Barcelona, Spain). Briefly, a sperm sample (2 μl) was placed in a pre-warmed (38 °C) Leja counting slide (Leja Products B.V., Nieuw-Vennep, The Netherlands), and 10 fields were analyzed at 38 °C to assess a minimum of 1000 spermatozoa per sample for total motile sperm (%) and progressive motile sperm (%).

### Sperm viability, acrosomal integrity, and intracellular ROS levels

Sperm cells (1 × 10^8^/ml) incubated for 2 h at 37.5 °C were washed twice with phosphate-buffered saline (PBS) containing 0.1% (w/v) polyvinyl alcohol (PBS-PVA). Sperm viability was assayed using a LIVE/DEAD® sperm viability kit (Molecular Probes, Eugene, OR, USA), which contained the DNA dyes SYBR14 (final conc. 100 nM) and propidium iodide (PI; final conc. 10 μM), according to the manufacturer’s instructions. To assess acrosomal integrity, sperm were stained with 10 μg/ml lectin peanut agglutinin-FITC conjugate (PNA) and PI, and images were then acquired using a fluorescence microscope (Eclipse Ci, Nikon Instruments Inc., Seoul, Korea) equipped with a camera (DS-Fi2, Nikon) and imaging software (version 4.30, Nikon). Spermatozoa were classified as viable (SYBR14 stained), dead (PI stained), or as intact (PNA+) or damaged (PNA−) acrosomal sperm. Intracellular ROS levels were assessed using 1 μM carboxy-DCFDA (Invitrogen, Eugene, OR, USA), and fluorescence intensities were measured using a multimode microplate reader (Spark™ 10 M, Tekan, Männedorf, Switzerland) at excitation (ex.) and emission (em.) wavelengths of 485 and 520 nm, respectively.

### Collection and in vitro maturation (IVM) of pig oocytes

Ovaries were collected from prepubertal gilts at a local slaughterhouse. Cumulus–oocyte complexes (COCs) were aspirated from antral follicles (3–6 mm in diameter), washed three times in HEPES-buffered Tyrode lactate (TL-HEPES-PVA) medium supplemented with 0.01% (w/v) PVA, and then washed three times with oocyte maturation medium (Abeydeera et al. 1998). A total of 50 COCs were transferred to 500 µl of maturation medium and layered with mineral oil in a 4-well multi-dish equilibrated at 38.5 °C in 5% CO_2_ in air. The oocyte maturation medium used was tissue culture medium (TCM) 199 supplemented with 0.1% PVA, 3.05 mM D-glucose, 0.91 mM sodium pyruvate, 0.57 mM cysteine, 0.5 µg/ml luteinizing hormone, 0.5 µg/ml follicle-stimulating hormone, 10 ng/ml epidermal growth factor, 75 µg/ml penicillin G, and 50 µg/ml streptomycin. Oocytes were cultured in TCM199 for 44 h at 38.5 °C, 5% CO_2_ in air.

### In vitro fertilization (IVF) and culture (IVC) of pig oocytes

After IVM, cumulus cells were removed by treating them with 0.1% hyaluronidase in TL-HEPES-PVA medium (Abeydeera et al. 1998). Oocytes were then placed into four 100 μl drops of modified Tris-buffered medium (mTBM) in a 35-mm polystyrene culture dish and covered with mineral oil. Spermatozoa were incubated in BTS in the absence (W/O) or presence of TiO_2_ NRs (controls), Au-TiO_2_ NRs, or Ag-TiO_2_ NRs (final conc.: 10 or 20 μg/ml) for 2 h at 38 °C and washed twice in PBS containing 0.1% PVA (PBS-PVA) at 800× *g* for 5 min. At the end of the washing procedure, sperm were resuspended in mTBM, appropriately diluted, and sperm suspensions (1 µl) were added to medium containing oocytes to a final sperm concentration of 1 × 10^5^ spermatozoa/ml. Oocytes were co-incubated with spermatozoa for 5 h at 38.5 °C in a 5% CO_2_ atmosphere. After IVF, oocytes were transferred to 500 μl porcine zygote medium (PZM-3; Yoshioka et al. 2002), supplemented with 0.4% bovine serum albumin, and cultured for an additional 20, 48, or 144 h. The IVM, IVF, and IVC studies were repeated five times for each treatment regimen.

### Fluorescence staining of oocytes and spermatozoa

Oocytes/embryos were fixed with 2% formaldehyde for 40 min at room temperature (RT), washed twice with PBS, permeabilized with PBS-Triton X-100 for 30 min, and stained with 2.5 mg/ml 4′,6-diamidino-2-phenylindole (DAPI; DNA staining; Molecular Probes, Eugene, OR, USA) for 40 min. The fertilization statuses of zygotes (unfertilized, fertilized-monospermic, or fertilized-polyspermic), cleaved embryo numbers, blastocyst formation, cell number per blastocyst were determined under a fluorescence microscope (Nikon Eclipse Ci microscope; Nikon Instruments Inc., Seoul, Korea). To observe the attachment of TiO_2_ NRs to spermatozoa, TiO_2_ NRs (1 mg/ml) were mixed with 1 mM alizarin red S (ARS) and stored at 4 °C until required (Thurn et al. 2009). Spermatozoa (1 × 10^8^/ml) were incubated in BTS in the presence of TiO_2_ NRs-ARS for 2 h at 38.5 °C. Spermatozoa were then fixed with 2% formaldehyde for 40 min at RT, washed with PBS three times, stained with 2.5 μg/ml DAPI and 10 μg/ml PNA for 40 min, and then observed under a fluorescence microscope (Nikon).

### Observations of sperm incubated with TiO_2_ NRs by TEM

Spermatozoa incubated with TiO_2_ NRs, Ag-TiO_2_ NRs, or Au-TiO_2_ NRs were fixed in modified Karnovsky’s fixative (2% paraformaldehyde and 2% glutaraldehyde in 0.05 M sodium cacodylate buffer (pH 7.2) at 4 °C overnight, washed three times with 0.05 M sodium cacodylate buffer at 4 °C for 10 min, and postfixed in 1% osmium tetroxide in 0.05 M sodium cacodylate buffer for 90 min. Fixed cells were washed twice with DI water at RT and stained using 0.5% uranyl acetate at 4 °C overnight. Further, fixed cells were dehydrated using an increasing ethanol series (30, 40, 50, 70, 80, 90, and 100%), embedded in EMbed 812 resin mixture containing DDSA, NMA, and DMP-30, polymerized at 60 °C for 48 h, and ultra-thin sections were stained with 2% uranyl acetate for 45 min followed by lead citrate for 3 min. TEM was conducted using a Hitachi H-7650 unit at 80 kV (Tokyo, Japan).

### Statistical analysis

All experimental data were expressed as mean ± standard error of the mean (SEM), and analyzed using one-way ANOVA in GraphPad PRISM® (GraphPad software, San Diego, CA, USA). The completely randomized design was applied, and Tukey’s multiple comparison test was performed to compare values of individual treatments. Results are considered statistically significant at *p < 0.05, **p < 0.01 and ***p < 0.001.

## Results

### Characterizations of noble metal-loaded TiO_2_ NRs

**Fig. 1:**
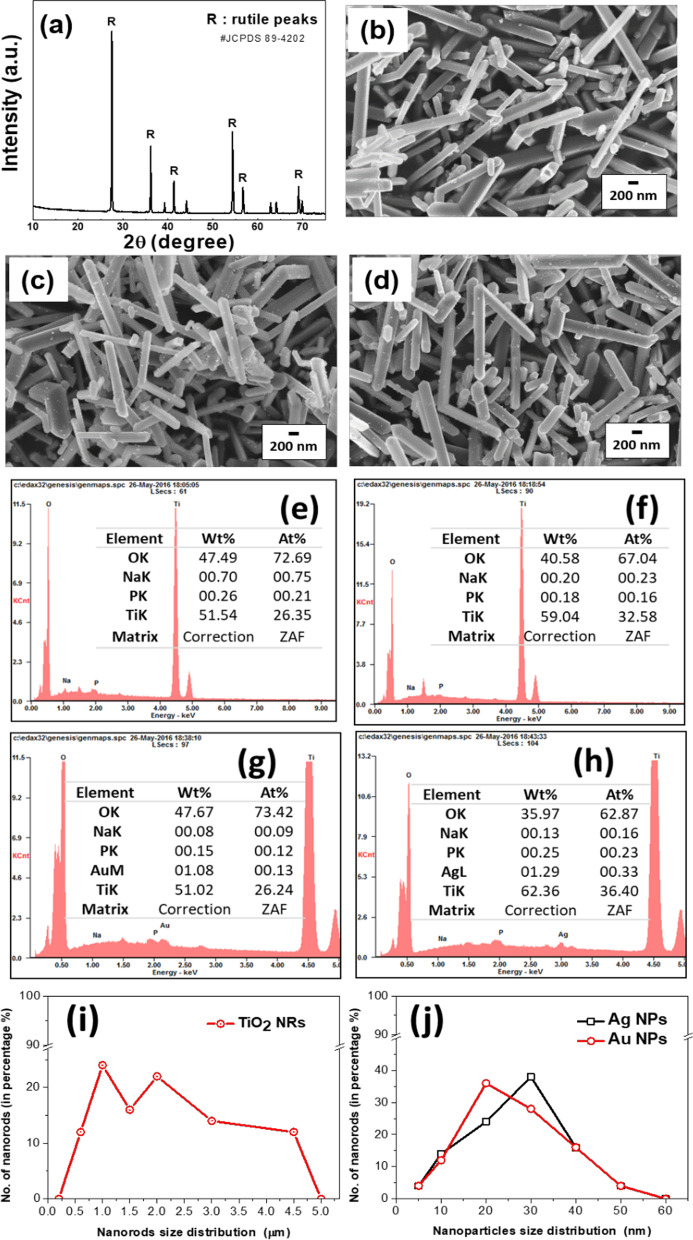
Characteristics of novel metal-loaded TiO_2_ NRs. **A** XRD image of rutile TiO_2_ NRs. FESEM images of **B** bare TiO_2_ NRs, **C** Ag-loaded TiO_2_ NRs, and **D** Au-loaded TiO_2_ NRs. EDS spectrum and elemental quantification table (inset) of **E** TiO_2_-NRs, **F** 2-time washed TiO_2_-NRs, **G** Au (1wt%)-TiO_2_-NRs, and **H** Ag (1wt%)-TiO_2_-NRs. Particle size (length) distribution histogram for **I** TiO_2_ NR, and **J** deposited noble metals (Au and Ag)

The XRD patterns of TiO_2_ NRs are shown in Fig. 1A. Major diffraction peaks at 2*θ* = 27.5, 36.1, and 54.4° correspond to (110), (101), and (211) crystal planes, which is the most reported phase of rutile (JCPDS 89-4202) (Dhandole et al. 2016). However, small length NRs (or broken residuals) were also observed which contributed small portion in the overall synthesized product. The elemental analysis EDS is shown in Fig. 1E–H and represents the elemental quantifications and noble metals (1 wt%) observed on the surface of TiO_2_ NRs. Particle size distribution histogram for TiO_2_ NR and deposited noble metals (Au and Ag) were illustrated in Fig. 1I, J. Ag and Au particle size was determined by TEM analysis.

**Fig. 2:**
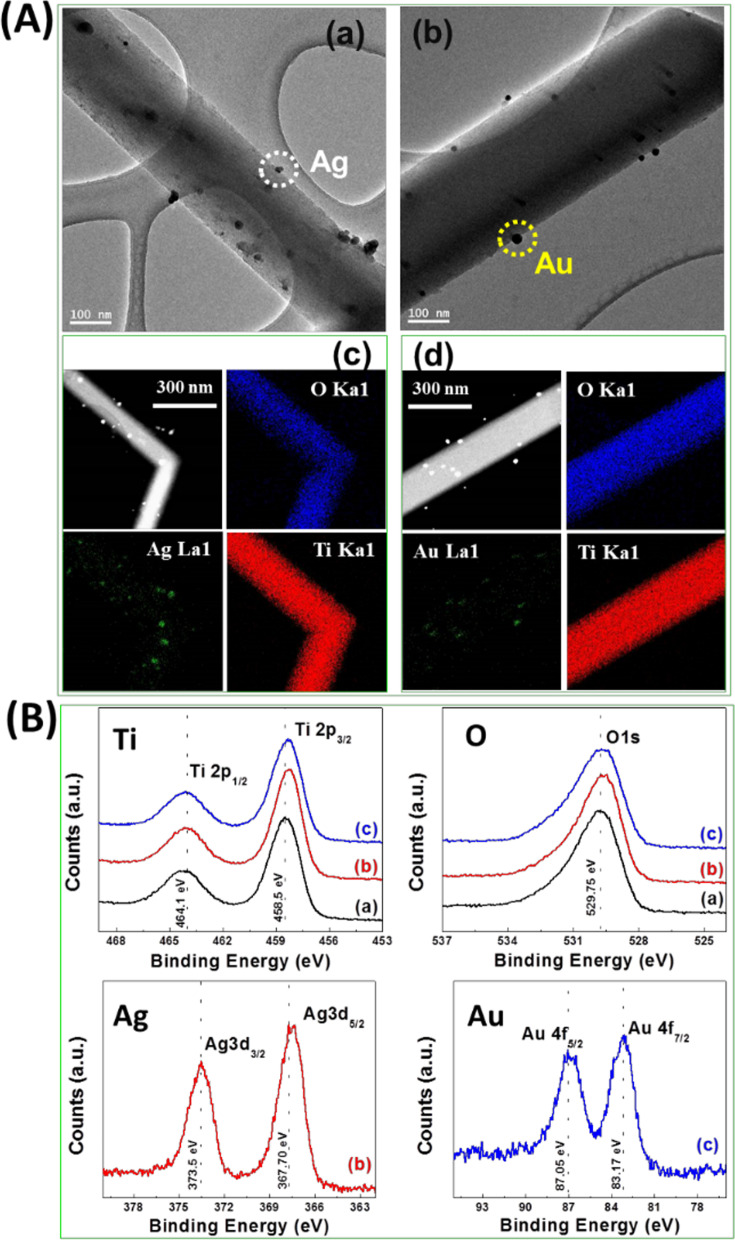
Nanoparticle attachment to TiO_2_ NRs as determined by electron microscopy. **A** TEM images of (a) silver (Ag) nanoparticle-loaded TiO_2_ NRs and (b) gold (Au) nanoparticle-loaded TiO_2_ NRs. Dotted circles show Ag and Au nanoparticles on the surfaces of TiO_2_ NRs. (c) and (d) show respective TEM maps. **B** High-resolution XPS spectra of Ti 2p, O 1s, Ag 3d, and Au 4f oxidation peaks of (a) bare TiO_2_ NRs, (b) Ag-loaded TiO_2_ NRs, and (c) Au-loaded TiO_2_ NRs

As shown in Fig. 2A (a, b), Ag and Au NPs were observed on the surfaces of TiO_2_ NRs and both had a diameter of 20–30 nm as determined by TEM. The oxidation states of elements and the chemical compositions of the as-synthesized materials were determined by XPS. Figure 2B shows the high-resolution XPS spectra for Ti 2p and O 1s of TiO_2_ NRs and the noble metal-loaded TiO_2_ NRs. XPS peaks at around 458.5 and 464.0 eV correspond to Ti 2p_3/2_ and Ti 2p_1/2_ spin–orbit pairs, respectively, confirming that titanium doublet peaks were due to the Ti (IV) oxidation state (Li et al. 2005; Ohno et al. 2003). The peak at ~ 529 eV was ascribed to oxygen. The shifts of Ag and Au peaks to lower energies compared with bulk material confirmed large quantities of both metals deposited on the surfaces of TiO_2_ NRs and that strong metallic interactions had formed between noble metal NPs and the TiO_2_ NRs. The XPS spectra of Ag 3d and Au 4f contained doublet peaks located at ~ 367.7 and ~ 373.5 eV, which corresponded to the reported binding energies of Ag 3d_5/2_ and Ag 3d_3/2_, respectively, and peaks at ~ 83.0 and ~ 87.05 eV, which corresponded to the binding energies of Au 4f_7/2_ and Au 4f_5/2_, respectively (Su et al. 2012; Zhang et al. 2014; Haruta 1997; Ma et al. 2014).

### Sperm motility in the presence of noble metal-loaded TiO_2_ NRs

**Fig. 3:**
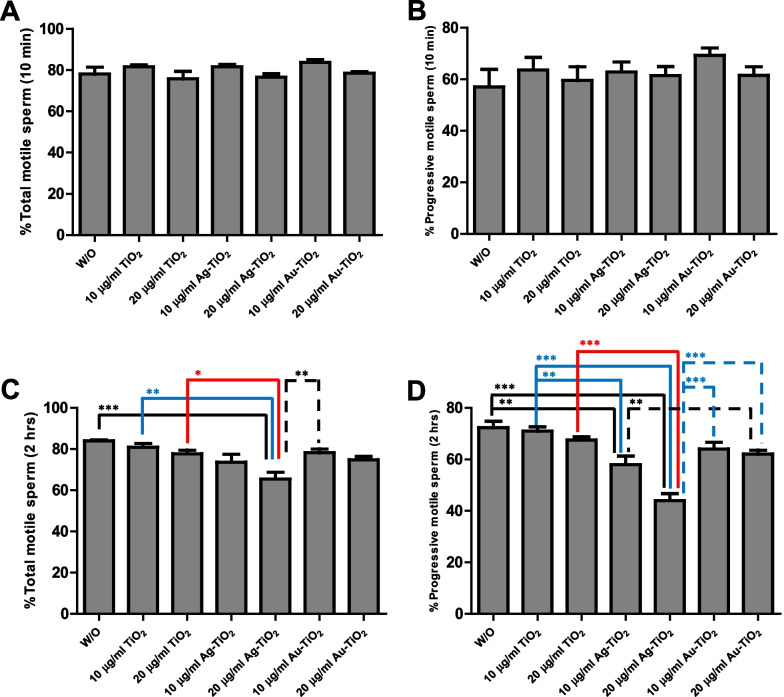
Comparison of total motile sperm and progressive motile sperm after incubation with or without (W/O) TiO_2_ NRs. Boar spermatozoa were incubated in the presence of TiO_2_ NRs for 10 min (**A**, **B**) and 2 h (**C**, **D**). Values are expressed as mean ± SEM. Lines (or dotted lines) among columns denote significant differences at *p < 0.05, **p < 0.01 and ***p < 0.001

We assessed sperm motilities using a CASA system; motility parameters after treatments for 10 min and 2 h are provided in Fig. 3. The percentage of total motile sperm after 10 min of incubation was higher for sperm treated with 10 μg/ml of Au-TiO_2_ NRs than for sperm treated with 20 μg/ml of Ag-TiO_2_ NRs; however, differences between treatment groups were not significant (Fig. 3A, B). After incubation for 2 h, motility was significantly greater for non-treated controls than in the other groups, and the motility of sperm incubated with 20 μg/ml of Ag-TiO_2_ NRs was significantly less than in the other groups (*p < 0.05, **p < 0.01 and ***p < 0.001; Fig. 3C). The percentage of progressive motile spermatozoa was higher after treatment with 10 μg/ml Au-TiO_2_ NRs for 10 min, but then decreased after treatment for 2 h. In particular, we observed significant progressive loss of motility after treatment with 20 μg/ml Ag-TiO_2_ NRs (**p < 0.01 and ***p < 0.001; Fig. 3D). Motility refers to the ability of spermatozoa to move and swim independently in the female reproductive tract and is essential for successful fertilization. These results suggest that Ag-TiO_2_ NRs affects sperm movement either physically or chemically.

### Sperm viability, acrosomal integrity, and ROS levels in spermatozoa incubated with TiO_2_ NRs

**Fig. 4:**
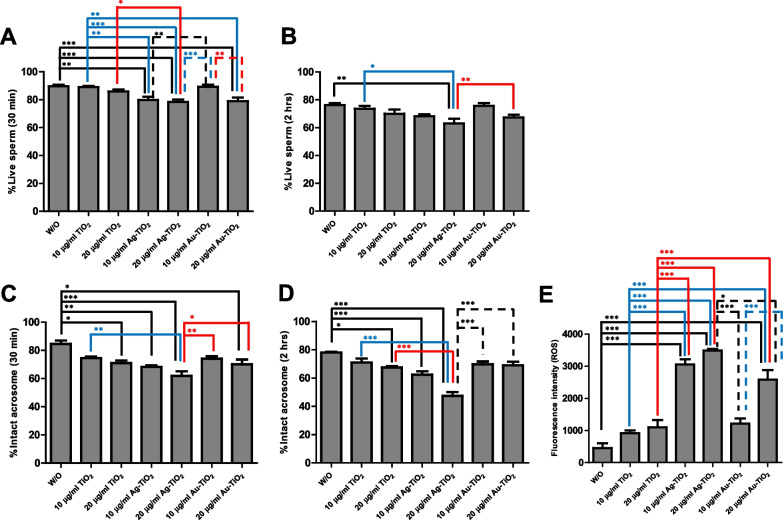
Assessment of sperm viability and acrosomal integrity. Boar spermatozoa were incubated in the absence (W/O) or presence of NRs. Live (viable) spermatozoa were counted after incubation for 30 min (**A**) or 2 h (**B**). Also, intact acrosomes were examined using PNA and PI staining after incubation for 30 min (**C**) or 2 h (**D**). **E** Intracellular ROS generation in spermatozoa exposed to TiO_2_ NRs. Fluorescence intensities were measured in sperm stained with carboxy-DCFDA. Experiments were repeated five times with spermatozoa from two different boars. Values are expressed as mean ± SEM. Lines (or dotted lines) among columns denote significant differences at *p < 0.05, **p < 0.01, and ***p < 0.001

Figure 4 shows viability and intact acrosome results for sperm incubated for 30 min or 2 h with TiO_2_ NRs, Ag-TiO_2_ NRs, or Au-TiO_2_ NRs. Percentages of viable sperm were higher after treatment with 10 μg/ml Au-TiO_2_ NRs and for controls than after treatment with 20 μg/ml Au-TiO_2_ NRs 10 or 20 μg/ml Ag-TiO_2_ NRs (75.7–76.3% vs. 63.0–73.6%; *p* < 0.05, *p* < 0.01 and *p* < 0.001; Fig. 4A). These results indicate that Ag-TiO_2_ NRs at 10 and 20 μg/ml adversely affected the viability of spermatozoa, and the higher decrement obtained for 2 h incubation signifies that sperm movement might be disturbed under high doses of Ag-TiO_2_ NRs (*p* < 0.05 and *p* < 0.01; Fig. 4B). Regarding acrosomal integrity, there was a high percentage of intact acrosome spermatozoa (PNA-/PI-) in the no treatment sample, but damaged acrosomes significantly increased in the 20 μg/ml Ag-TiO_2_ NR group after incubation of 30 min or 2 h than in the other groups (*p* < 0.05, *p* < 0.01 and *p* < 0.001; Fig. 4C, D). To understand the effects of catalysts on the motility and viability of spermatozoa, we measured intracellular ROS levels after treating sperm for 2 h. Interestingly, ROS levels were found to be significantly higher after treatment with Ag-TiO_2_ NRs than for the other treatments (*p* < 0.05 and *p* < 0.001; Fig. 4E).

**Fig. 5:**
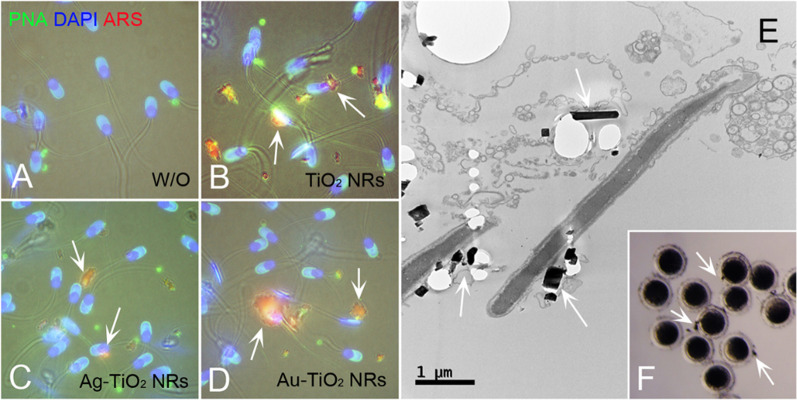
Spermatozoa were incubated with alizarin red S (ARS, red), and stained with PNA (sperm acrosome, green) and DAPI (DNA, blue). **A** Control (without [W/O] catalyst), **B** TiO_2_ NRs, **C** Ag-loaded TiO_2_ NRs, and **D** Au-loaded TiO_2_ NRs for 4 h. **E** TEM image of TiO_2_ NRs attached to sperm acrosome and nuclear membrane (white arrows). **F** TiO_2_ NRs attached to spermatozoa inhibited sperm penetration during IVF

Spermatozoa were incubated with ARS-coated TiO_2_ NRs (controls, Ag-loaded, and Au-loaded) for 4 h, washed twice with PBS-PVA, fixed, and stained with PNA (sperm acrosome, green) and DAPI (DNA, blue; A–D); all NRs showed sufficient ARS (red fluorescence; Fig. 5). Scattered or clumped coated TiO_2_ NRs (stained red) were attached to sperm heads, midsections, or tails (white arrows) after each treatment (Fig. 5A–D). In TEM analysis, Ag-TiO_2_ NRs and nanoparticles were observed in acrosomal membrane (white arrows; Fig. 5E) (Lan and Yang 2012; Lafuente et al. 2016; Li et al. 2005, 2014; Ma et al. 2014). TiO_2_ NRs attached to spermatozoa were also observed during IVF, which presumably would prevent sperm penetrating oocytes (Fig. 5F).

### Fertilization and embryo development rates on sperm incubated with TiO_2_ NRs

**Fig. 6:**
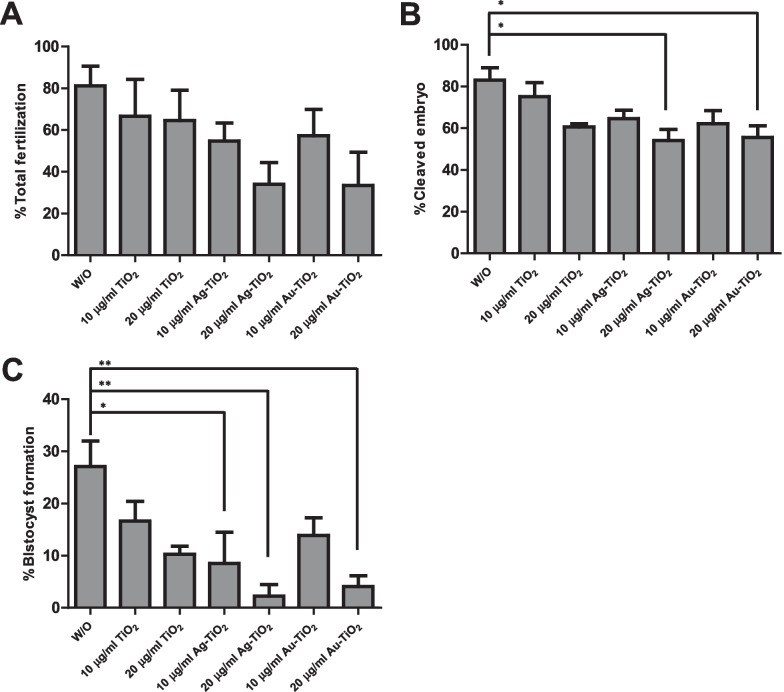
Fertilization competence of sperm incubated with/without TiO_2_ NRs. **A** Oocytes were fertilized with sperm incubated with control (W/O), Ag-loaded, and Au-loaded TiO_2_ NRs. **B** After IVF, fertilized oocytes were cultured for 144 h to observe subsequent embryo development. Numbers of inseminated oocytes are indicated in parentheses. Experiments were independently performed five times. Values are expressed as mean ± SEM. Lines (or dotted lines) among columns denote significant differences at *p < 0.05 and **p < 0.01

To determine fertilization rates, oocytes were inseminated with spermatozoa incubated with controls and TiO_2_ NRs, Au-TiO_2_ NRs, or Ag-TiO_2_ NRs for 2 h (Fig. 6A). The total fertilization rate including the rate of monospermic and polyspermic oocytes was lower when spermatozoa were incubated with 20 μg/ml Ag-TiO_2_ NRs (monospermic: 33.9%, polyspermy: 0%) or 20 μg/ml Au-TiO_2_ NRs (monospermic: 33.4%, polyspermy: 0%) than controls (monospermic: 46.9–64.4%, polyspermy: 12.4–16.7%) and other treatments (monospermic: 41.5–54.8%, polyspermy: 0–15.8%), but there were no significantly differences among groups (Fig. 6A). As regards embryonic development, we observed more cleaved oocytes from IVF performed using control sperm (83.1%) and a significantly lower cleavage rate when sperm were incubated with 20 μg/ml Ag-TiO_2_ NRs (54.1%) or 20 μg/ml Au-TiO_2_ NRs (55.6%) than in the other groups (60.6–75.1%, *p* < 0.05; Fig. 6B). Furthermore, the blastocyst formation rate was higher in the control group (27.1%) than in the other groups (10.2–4.1%; Fig. 6C), and when oocytes were fertilized with spermatozoa incubated with 20 μg/ml Ag-loaded TiO_2_ NRs, the lowest blastocyst rates were observed (2.2%, *p* < 0.05 and *p* < 0.001; Fig. 6C). However, mean cell number per blastocyst in the groups were not significantly different (30.1–45.0 cells/blastocyst).

### Intracellular ROS generation by Ag-TiO_2_ NRs in spermatozoa

**Fig. 7:**
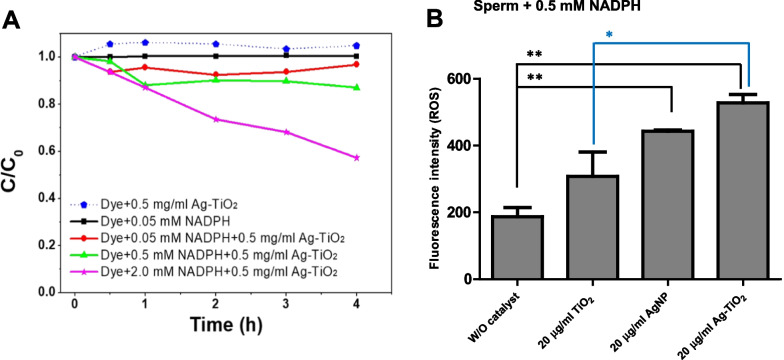
ROS generation in spermatozoa incubated with Ag-loaded TiO_2_ NRs using NADPH. **A** Dye degradation of Ag-loaded TiO_2_ NRs in the presence of NADPH. **B** ROS generation (in sperm) incubated with different catalysts Ag nanoparticles, TiO_2_ NRs, and Ag-TiO_2_ NR in the presence of commercial NADPH. Values are expressed as mean ± SEM. Lines (or dotted lines) among columns denote significant differences at *p < 0.05 and **p < 0.01

**Fig. 8:**
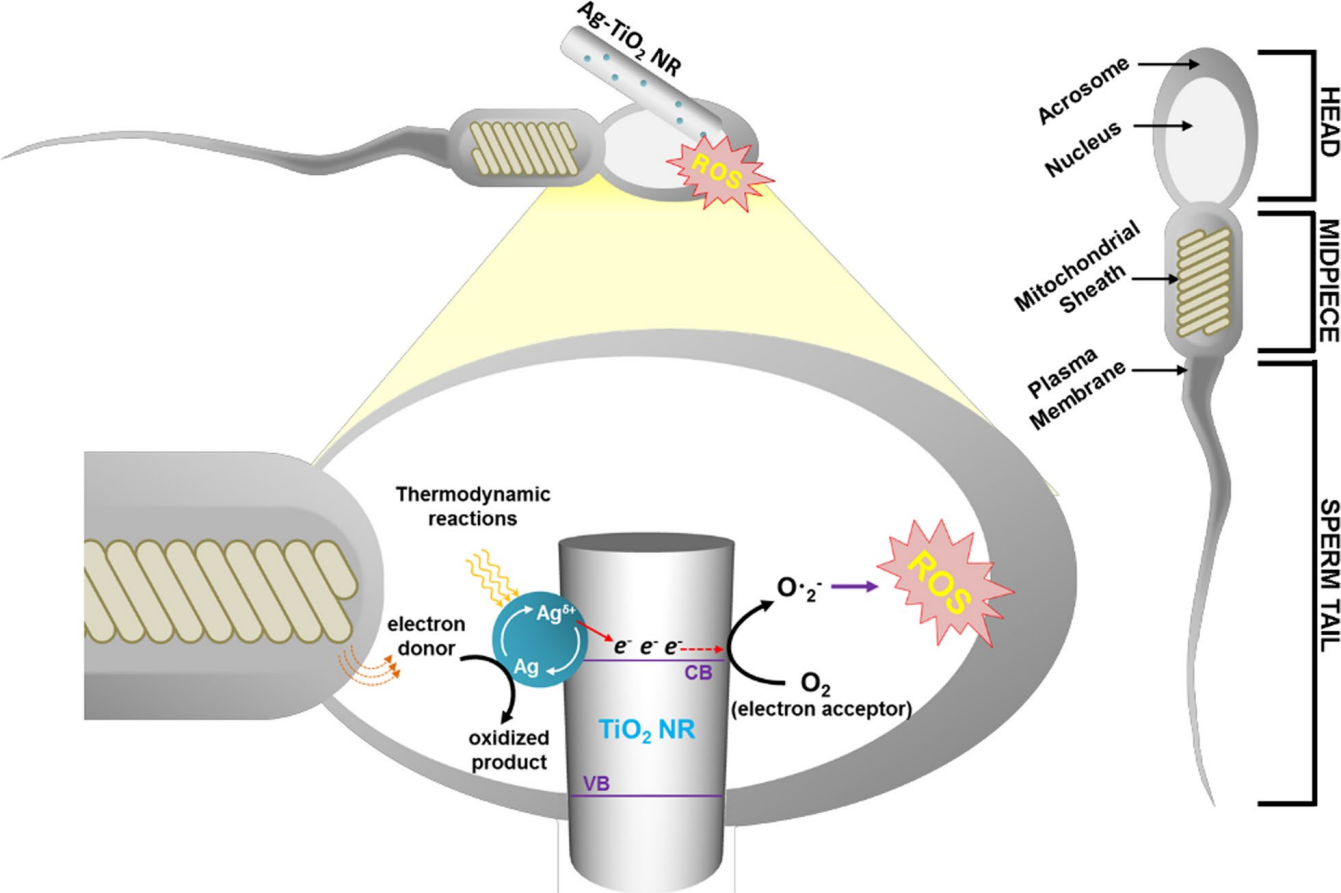
Schematic representation of electron transfer mechanism in boar spermatozoa incubated with Ag-loaded TiO_2_ NR catalyst

To investigate intracellular ROS generation in sperm exposed to Ag-TiO_2_ NR, we first examined the role of NADPH during the process of electron donation. Specifically, we tested the effects of NADPH activity over the degradation of organic orange II dye in the presence of Ag-TiO_2_ NR catalyst. Orange (II) dye contains an azo linkage, which forms N=N bonds with its chromophores benzene and naphthalene, and this linkage is sensitive to active radical species such as OH·, HOO·, and O·_2_^−^ (Dhandole et al. 2017). We observed low dye concentrations in the presence of 0.05–2 mM NADPH and 0.5 mg/ml Ag-TiO_2_ NRs (Fig. 7A), but single treatments with NADPH or Ag-loaded TiO_2_ NRs had no effect. This result indicates that the synergistic effect of NADPH and Ag-TiO_2_ NR treatment for 4 h increased the dye degradation rate as increasing concentration of NADPH (Fig. 7A). Based on this result, we fixed the concentration of NADPH at 0.2 mM and conducted the same experiment but replaced the organic dye with fresh sperm (Fig. 7B). We observed higher ROS levels when NADPH (0.2 mM) and Ag-loaded TiO_2_ NRs were treated in combination; ROS production was low when only NADPH was administered (Fig. 7B). These results indicate the importance of NADPH as an electron donor (Aitken et al. 1997). In the presence of low NADPH levels, Ag-loaded TiO_2_ NRs show enhanced generating of ROS, which suggests these NRs acted as charge carriers from NADPH donor ligands (Fig. 8).

## Discussion

Remarkable advances in nanotechnology have made many novel biomedical applications possible, especially in the reproductive biology field. However, due to the environmental effects of the widespread use of NPs and their adverse effects on animal germ cells, their use should be subjected to strict review (Falchi et al. 2018). In particular, NPs have been recently reported to be toxic to male reproductive organs and germ cells. Small NPs easily penetrate cell membranes, such as the blood–testis barrier, accumulate or deposit in testis, and disrupt sperm formation and development (Lan and Yang 2012). In rats fed Ag NPs, sex hormone levels decreased and abnormal sperm morphology and motility increased (Neill et al. 2009; Baki et al. 2014; Lafuente et al. 2016), and administering Au NPs to mice caused abnormal sperm chromatin remodeling and DNA damage (Nazar et al. 2016). In similar experiments, functional defects and DNA damage in spermatozoa were observed in male mice administered TiO_2_ NPs (Smith et al. 2015), and direct exposure to NPs interfered with sperm function, for example, bull sperm exposed to Au NPs showed impaired motility and fertility (Taylor et al. 2014), and human sperm treated with 44 ppm of Au NPs showed 25% lower motility than non-treated controls (Wiwanitkit et al. 2009). In the present study, direct exposure of Ag-loaded TiO_2_ NRs to boar spermatozoa decreased motility, viability, and acrosomal integrity, which resulted in lower fertilization and embryonic development rates.

Spermatozoa are highly sensitive cells dedicated to fertilizing oocytes, and many factors can influence sperm attachment and fertilization. The most problematic factor is the intracellular production of ROS and related oxidative stress, which adversely affects sperm survival and fertility (Saadeldin et al. 2020). Vernet et al. (2001) suggested that intracellular ROS generation might be a collective effect of the mitochondrial respiratory chain and NADPH oxidase system in sperm plasma membranes. The mitochondrial electron transport chain reaction produces ROS under physiological conditions, and this is correlated with physical activity (Riaz et al. 2017; Vernet et al. 2004). However, recent studies have confirmed that the cytotoxic effect of Ag NPs can also induce intracellular ROS production, which is responsible for most of the common abnormalities in spermatozoa, such as disrupted chromatin, bent tails, curved midsections, DNA fragmentation, mitochondrial damage, respiratory chain disruption, oxidative stress, and chromosomal aberrations (Haase et al. 2012; Beer et al. 2012; Wang et al. 2017).

Based on a literature review, we report an electron transfer mechanism that underlies spermatozoa inactivation and present for the first time the effect metal oxide nanoparticles have on intracellular ROS stress in spermatozoa caused by mitochondrial transmembrane electron transport. We present the suggested general electron transfer mechanism in a schematic diagram in Fig. 8. The physical contact of sperm cell with Ag-loaded TiO_2_ NR will result in Schottky barrier whose Fermi-level alignment (based on band theory) results in the facile electron transport from spermatozoa to Ag NPs over the surface of TiO_2_ NRs. The electron donor ligand of Ag NP might accept the transmembrane electrons of sperm enzymatic NADPH after mitochondrial electron transport chain reactions have been disrupted. The affinity of the Ag^+^ metallic state is higher than the donor ligand, and reduction potential is a parameter of thermodynamic reactions (Vernet et al. 2001; Prasad et al. 2017). The reduced species, Ag^0^, transfer electrons to the conduction band of TiO_2_ because the band edge of the TiO_2_ is lower than that of the Ag-loaded NPs (as shown in Fig. 7; Kochuveedu et al. 2013). After an electron is released, Ag re-oxidizes, and the cycle continues until the donor ligand actively participates in the reaction. Meanwhile, conduction band electrons reduce oxygen molecules to form OH·, O_2_^−^, and OOH· radicals (Dhandole et al. 2017), which cause cell damage and DNA fragmentation. In a recent study, it was reported that morin and rutin have protective effects on rat testis from oxidative damage caused by ROS associated with TiO_2_ NP intake (Hussein et al. 2019). Therefore, a further study on antioxidants to mitigate the toxicity and oxidative damage associated with ROS production derived from Ag- or TiO2 NRs is needed in the future.

## Conclusions

In the present study, Ag or Au NPs were incorporated on the surfaces of TiO_2_ NRs by photo-deposition, and their effects on boar spermatozoa were examined. In addition, porcine oocytes matured in vitro were inseminated with sperm that had been incubated in the presence of the noble metal-loaded NRs, and fertilization rates and embryo developments were investigated to verify germ cell toxicity in preimplantation embryos. Our experimental results indicated that Ag-TiO_2_ NRs at 20 μg/ml generated ROS that decreased the fertilization rate and reduced sperm viability and acrosome integrity. Furthermore, our investigation of the charge transfer mechanism and ROS generation showed that a catalytic redox reaction took place in sperm nuclei and that mitochondrial NADPH functions as an electron donor and activates Ag-loaded TiO_2_ NRs.

## Data Availability

Upon reasonable request, the datasets of this study can be available from the corresponding author.

## References

[CR1] Abeydeera LR, Wang W, Prather RS, Day BN (1998). Maturation in vitro of pig oocytes in protein-free culture media: fertilization and subsequent embryo development in vitro. Biol Reprod.

[CR2] Aitken RJ, Fisher HM, Fulton N, Gomez E, Knox W, Lewis B, Irvine S (1997). Reactive oxygen species generation by human spermatozoa is induced by exogenous NADPH and inhibited by the flavoprotein inhibitors diphenylene iodonium and quinacrine. Mol Reprod Dev.

[CR3] Baki ME, Miresmaili SM, Pourentezari M, Amraii E, Yousefi V, Spenani HR, Talebi AR, Anvari M, Eazilati M, Fallah AA, Mangoli E (2014). Effects of silver nano-particels on sperm parameters, number of Leydig cells and sex hormones in rats. Iran J Reprod Med.

[CR4] Beer C, Foldbjerg R, Hayashi Y, Sutherland D, Autrup H (2012). Toxicity of silver nano-particles- nanoparticle or silver ion?. Toxicol Lett.

[CR5] Chang X, Zhang Y, Tang M, Wang B (2013). Health effects of exposure to nano-TiO_2_: a meta-analysis of experimental studies. Nanoscale Res Lett.

[CR6] Cornish BJPA, Lawton LA, Robertson PKA (2000). Hydrogen peroxide enhanced photocatalytic oxidation of microcystin-LR using titanium dioxide. Appl Catal B.

[CR7] Czajka M, Sawicki K, Sikorska K, Popek S, Kruszewski M, Kapka-Skrzypczak L (2015). Toxicity of titanium dioxide nanoparticles in central nervous system. Toxicol In Vitro.

[CR8] Dhandole LK, Ryu J, Lim JM, Oh BT, Park JH, Kim BG, Jang JS (2016). Hydrothermal synthesis of titanate nanotubes from TiO_2_ nanorods prepared via a molten salt flux method as an effective adsorbent for strontium ion recovery. RSC Adv.

[CR9] Dhandole LK, Mahadik MA, Kim SG, Chung HS, Seo YS, Cho M, Ryu J, Jang JS (2017). Boosting photocatalytic performance of inactive rutile TiO_2_ nanorods under solar light irradiation: synergistic effect of acid treatment and metal oxide co-catalysts. ACS Appl Mater Interfaces.

[CR10] Dhandole LK, Kim S, Seo Y, Mahadik M, Chung H, Lee S, Choi S, Ryu J, Jang JS (2018). Enhanced photocatalytic degradation of organic pollutants and inactivation of *Listeria monocytogenes* by visible light active Rh−Sb co-doped TiO_2_ nanorods. ACS Sustain Chem Eng.

[CR11] Falchi L, Khalil WA, Hassan M, Marei WFA (2018). Perspectives of nanotechnology in male fertility and sperm function. Int J Vet Sci Med.

[CR12] Gao GD, Ze YG, Li B, Zhao XY, Zhang T, Sheng L, Hu RP, Gui SX, Sang XZ, Sun QQ, Cheng J, Cheng Z, Wang L, Tang M, Hong FS (2012). The ovarian dysfunction and its gene-expressed characteristics of female mice caused by long-term exposure to titanium dioxide nanoparticles. J Hazard Mater.

[CR13] Guo LL, Liu XH, Qin DX, Gao L, Zhang HM, Liu JY, Cui YG (2009). Effects of nanosized titanium dioxide on the reproductive system of male mice. Zhonghua Nankexue.

[CR14] Haase A, Rott S, Mantion A, Graf P, Plendl J, Thünemann AF, Meier WP, Taubert A, Luch A, Reiser G (2012). Effects of silver nanoparticles on primary mixed neural cell cultures: uptake, oxidative stress and acute calcium responses. Toxicol Sci.

[CR15] Haruta M (1997). Size- and support-dependency in the catalysis of gold. Catal Today.

[CR16] Hussein MM, Gad E, Ahmed MM, Arisha AH, Mahdy HF, Swelum AA, Tukur HA, Saadeldin IM (2019). Amelioration of titanium dioxide nanoparticle reprotoxicity by the antioxidants morin and rutin. Environ Sci Pollut Res.

[CR17] Kochuveedu ST, Jang YH, Kim DH (2013). A study on the mechanism for the interaction of light with noble metal-metal oxide semiconductor nanostructures for various photophysical applications. Chem Soc Rev.

[CR18] Lafuente D, Garcia T, Blanco J, Sanchez DJ, Sirvent JJ, Domingo JL (2016). Effects of oral exposure to silver nanoparticles on the sperm of rats. Reprod Toxicol.

[CR19] Lan Z, Yang WX (2012). Nanoparticles and spermatogenesis: how do nanopartilces affect spermatogenesis and penetrate the blood-testis barrier. Nanomedicine.

[CR20] Li D, Haneda H, Labhsetwar NK, Hishita S, Ohashi N (2005). Visible-light-driven photocatalysis on fluorine-doped TiO_2_ powders by the creation of surface oxygen vacancies. Chem Phys Lett.

[CR21] Li J, Zhou H, Qian S, Liu Z, Feng J, Jin P, Liu X (2014). Plasmonic gold nanoparticles modified titania nanotubes for antibacterial application. Appl Phys Lett.

[CR22] Ma J, Guo X, Zhang Y, Ge H (2014). Catalytic performance of TiO_2_@Ag composites prepared by modified photodeposition method. Chem Eng J.

[CR23] Moretti E, Terzuoli G, Renieri T, Iacoponi F, Castellini C, Giordano C, Collodel G (2013). In vitro effect of gold and silver nanoparticles on human spermatozoa. Andrologia.

[CR24] Nazar M, Talebi AR, Sharifabad MH, Abbasi A, Khoradmehr A, Danafar AH (2016). Acute and chronic effects of gold nanopartilces on sperm parameters and chromatin structure in mice. Int J Reprod Biomed.

[CR25] Neill MO, Hutchison G, Malone E (2009). The effect of nano-particle exposure on the male reproductive system of mice. Int J Androl.

[CR26] Ohno T, Mitsui T, Matsumura M (2003). Photocatalytic activity of S-doped TiO_2_ photocatalyst under visible light. Chem Lett.

[CR27] Pawar K, Kaul G (2014). Toxicity of titanium oxide nanoparticles causes functionality and DNA damage in buffalo (Bubalus bubalis) sperm in vitro. Toxicol Ind Health.

[CR28] Prasad K, Lekshmi GS, Ostrikov K, Lussini V, Blinco J, Mohandas M, Vasilev K, Bottle S, Bazaka K, Ostrikov K (2017). Synergic bactericidal effects of reduced graphene oxide and silver nanoparticles against Gram-positive and Gram-negative bacteria. Sci Rep.

[CR29] Pursel V, Johnson L (1976). Frozen boar spermatozoa: methods of thawing pellets. J Anim Sci.

[CR30] Riaz KB, Nagy AM, Brown RP, Zhang Q, Malghan SG, Goering PL (2017). Silver nanoparticles: significance of physicochemical properties and assay interference on the interpretation of in vitro cytotoxicity studies. Toxicol In Vitro.

[CR31] Rupa AV, Manikandan D, Divakar D, Sivakumar T (2007). Effect of deposition of Ag on TiO_2_ nanoparticles on the photodegradation of Reactive Yellow-17. J Hazard Mater.

[CR32] Saadeldin IM, Khalil WA, Alharbi MG, Lee SH (2020). The current trends in using nanoparticles, liposomes, and exosomes for semen cryopreservation. Animals.

[CR33] Shi H, Magaye R, Castranova V, Zhao J (2013). Titanium dioxide nanoparticles: a review of current toxicological data. Part Fibre Toxicol.

[CR34] Sichel C, Tello J, Cara MD, Fernandez-Ibanez P (2007). Effect of UV solar intensity and dose on the photocatalytic disinfection of bacteria and fungi. Catal Today.

[CR35] Smith MA, Michael R, Aravindan RG, Dash S, Shah S, Galileo DS, Martin-DeLeon PA (2015). Anatase titanium dioxide nanoparticles in mice: evidence for induced structural and functional sperm defects after short-, but not long-, term exposure. Asian J Androl.

[CR36] Su C, Liu L, Zhang M, Zhang Y, Shao C (2012). Fabrication of Ag/TiO_2_ nanoheterostructures with visible light photocatalytic function via a solvothermal approach. Cryst Eng Commun.

[CR37] Taylor U, Barchanski A, Peterson S, Kues WA, Baulain U, Gamrad L, Sajti L, Barcikowski S, Rath D (2014). Gold nanoparticles interfere with sperm functionality by membrane adsorption without penetration. Nanotoxicology.

[CR38] Taylor U, Tiedemann D, Rehbock C, Kues WA, Barcikowski S, Rath D (2015). Influence of gold, silver and gold/silver alloy nanoparticles on germ cell function and embryo development. Beilstein J Nanotechnol.

[CR39] Thurn KT, Paunesku T, Wu A, Brown EMB, Lai B, Vogt S, Maser J, Aslam M, Dravid V, Bergan R, Woloschak G (2009). Labeling TiO_2_ nanoparticles with dyes for optical fluorescence microscopy and determination of TiO_2_-DNA nanoconjugate stability. Small.

[CR40] Traversa E, Di-Vona ML, Nunziante P, Licoccia S, Sasaki T, Koshizaki N (2000). Sol-gel preparation and characterization of Ag-TiO_2_ nanocomposite thin films. J Sol-Gel Sci Technol.

[CR41] Vernet P, Fulton N, Wallace C, Aitken RJ (2001). Analysis of reactive oxygen species generating systems in rat epididymal spermatozoa. Biol Reprod.

[CR42] Vernet P, Aitken RJ, Drevet JR (2004). Antioxidant strategies in the epididymis. Mol Cell Endocrinol.

[CR43] Wang E, Huang Y, Du Q, Sun Y (2017). Silver nanoparticle induced toxicity to human sperm by increasing ROS (reactive oxygen species) production and DNA damage. Environ Toxicol Pharmacol.

[CR44] Wiwanitkit V, Sereemaspun A, Rojanathanes R (2009). Effect of gold nanoparticles on spermatozoa: the first world report. Fertil Steril.

[CR45] Wu L, Yu Y, Song L, Zhi J (2015). M/TiO_2_ (M = Au, Ag) transparent aqueous sols and its application on polymeric surface antibacterial post-treatment. J Colloid Interface Sci.

[CR46] Yoisungnern T, Choi YJ, Han JW, Kang MH, Das J, Gurunathan S, Kwon DN, Cho SG, Park C, Chang WK, Chang BS, Parnpai R, Kim JH (2015). Internalization of silver nanoparticles into mouse spermatozoa results in poor fertilization and compromised embryo development. Sci Rep.

[CR47] Yoshioka K, Suzuki C, Tanaka A, Anas IM, Iwamura S (2002). Birth of piglets derived from porcine zygotes cultured in a chemically defined medium. Biol Reprod.

[CR48] Zhang X, Liu Y, Lee ST, Yang S, Kang Z (2014). Coupling surface plasmon resonance of gold nanoparticles with slow-photon-effect of TiO_2_ photonic crystals for synergistically enhanced photoelectrochemical water splitting. Energy Environ Sci.

[CR49] Zhang J, Suo X, Zhang J, Han B, Li P, Xue Y, Shi H (2016). One-pot synthesis of Au/TiO_2_ heteronanostructure composites with SPR effect and its antibacterial activity. Mater Lett.

